# Ethical, Stigma, and Policy Implications of Food Addiction: A Scoping Review

**DOI:** 10.3390/nu11040710

**Published:** 2019-03-27

**Authors:** Stephanie E. Cassin, Daniel Z. Buchman, Samantha E. Leung, Karin Kantarovich, Aceel Hawa, Adrian Carter, Sanjeev Sockalingam

**Affiliations:** 1 Department of Psychology, Ryerson University, 350 Victoria St., Toronto, ON M5B 2K3, Canada; stephanie.cassin@psych.ryerson.ca; 2 Centre for Mental Health, University Health Network, Network - Toronto General Hospital, 200 Elizabeth Street, 8th Floor, Toronto, ON M5G 2C4, Canada; samantha.leung@uhn.ca (S.E.L.); kar.kantarovich@gmail.com (K.K.); 3 Department of Psychiatry, University of Toronto, 250 College Street, Toronto, ON M5T 1R8, Canada; 4 University of Toronto Joint Centre of Bioethics, 155 College Street, Suite 754, Toronto, ON M5T 1P8, Canada; daniel.buchman@uhn.ca; 5 Bioethics Program and Krembil Brain Institute, Toronto Western Hospital, University Health Network, 399 Bathurst Street, Toronto, ON M5T 1P8, Canada; 6 Dalla Lana School of Public Health, University of Toronto, 155 College Street, 6th Floor, Toronto, ON M5T 1R8, Canada; 7 Bariatric Surgery Program, University Health Network - Toronto Western Hospital, 399 Bathurst Street, East Wing – 4th Floor, Toronto, ON M5T 2S8, Canada; 8 Department of Education, Centre for Addiction and Mental Health, 33 Russell Street, Toronto, ON M5S 2S1, Canada; aceel.hawa@gmail.com; 9 School of Psychological Sciences, Monash University, Melbourne, VIC 3181, Australia; adrian.carter@monash.edu; 10 UQ Centre for Clinical Research, University of Queensland, Herston, QLD 4029, Australia

**Keywords:** ethics, food addiction, health policy, stigma

## Abstract

The concept of food addiction has generated much controversy. In comparison to research examining the construct of food addiction and its validity, relatively little research has examined the broader implications of food addiction. The purpose of the current scoping review was to examine the potential ethical, stigma, and health policy implications of food addiction. Major themes were identified in the literature, and extensive overlap was identified between several of the themes. Ethics sub-themes related primarily to individual responsibility and included: (i) personal control, will power, and choice; and (ii) blame and weight bias. Stigma sub-themes included: (i) the impact on self-stigma and stigma from others, (ii) the differential impact of substance use disorder versus behavioral addiction on stigma, and (iii) the additive stigma of addiction plus obesity and/or eating disorder. Policy implications were broadly derived from comparisons to the tobacco industry and focused on addictive foods as opposed to food addiction. This scoping review underscored the need for increased awareness of food addiction and the role of the food industry, empirical research to identify specific hyperpalatable food substances, and policy interventions that are not simply extrapolated from tobacco.

## 1. Introduction

The average weight of Americans increased dramatically in the 1980s, and this trend did not discriminate based on age, gender, or race [1]. Kessler notes that one remarkable change that coincided with the dramatic weight increase was the availability of “hyperpalatable” foods, specifically those engineered to combine optimal amounts of sugar, fat, and salt [2]. Some authors likened these hyperpalatable foods to addictive drugs, such as cocaine [3]. Though obesity only began garnering serious medical and public attention in recent years, the idea that obesity is a result of a food addiction was, in fact, first posited during the 1940s and 1950s [4,5]. However, this early conceptualization of food addiction resulted in much societal debate as it intensified weight stigma and contributed to ineffective policy changes around obesity and its consequences [5]. Since then, views on obesity and food addiction have been evolving, though the concept of food addiction is still highly debated today.

Food addiction is not an official diagnosis in the Diagnostic and Statistical Manual of Mental Disorders (DSM-5) [6]; however, health care professionals, researchers, patients, and the general public use the term, and a questionnaire (Yale Food Addiction Scale; YFAS) has been developed to diagnose its purported core features [7]. Many similarities have been noted between substance use disorders (e.g., alcohol, nicotine) and food addiction, including consuming more of the substance than intended or over a longer period of time, preoccupation with the substance, craving or strong urge to use the substance, and continued consumption despite knowledge of adverse effects [8].

Food addiction can have negative impacts on individuals living with obesity. Several studies have found that individuals with obesity who meet criteria for food addiction (as measured by the YFAS) demonstrate greater levels of eating disorder psychopathology, poorer general and health-related quality of life, greater depressive symptoms, and higher scores on impulsivity and self-control measures [9,10,11]. Together, this underscores the importance of studying the relationship between food addiction and obesity.

A food addiction model proposes that some individuals are addicted to certain foods and feel driven to engage in weight promoting eating behaviors, such as binge eating or compulsive overeating, when exposed to “addictive” food substances [7,12,13]. Foods with added fat and refined carbohydrates have been shown to be consumed in a more addictive manner [13,14] and craved more intensely [15] than less refined foods. This is thought to be, in part, a result of such hyperpalatable foods activating the mesolimbic reward-related pathway of the brain, which connects the ventral tegmental area of the midbrain to the nucleus accumbens in the ventral striatum via dopaminergic neurons [16,17]. Neuroimaging studies on individuals meeting the cut-off for food addiction according to the YFAS have been shown to have dysfunctional patterns of reward-related neural activation [12,18], and studies employing the use of genotyping have demonstrated higher multi-locus genetic profile scores in this group, which is associated with enhanced dopamine signaling [19].

Despite mounting evidence for the compulsive consumption of highly palatable foods, food addiction has continued to generate much controversy [20]. Discourses have centered around several issues, including diagnostic challenges of food addiction; arguments that food addiction is not distinct from binge eating disorder [21,22]; debates as to whether food addiction is akin to a behavioral addiction versus a substance use disorder [23]; and the notion that food (albeit not hyperpalatable foods), unlike other substances, is necessary for survival [24]. With respect to the diagnostic challenges, there is emerging evidence that food addiction is distinct from binge eating disorder [25] and may be best classified as a substance (food) use disorder as opposed to a behavioral (eating) addiction [8,26]. A recent systematic review examining the validity of food addiction concluded that the existing research supports food addiction as a unique construct [8]. Amongst the 35 articles (52 studies), the largest number of studies found evidence for brain reward dysfunction and impaired control, followed by studies on tolerance and withdrawal for foods with the greater addictive potential, specifically added sugars and fats.

In comparison to research examining the construct of food addiction and its validity, relatively little research has examined the potential ethical, stigma, and health policy implications of food addiction. Other addictions to substances, such as tobacco, cannabis, or alcohol, have seen an increase in the use of stigmatization as a policy tool in order to promote public health [27]. As a result, ethical issues have arisen surrounding public health communication and its effect on individuals and on society as a whole [28]. Bayer has argued that the use of stigma may reduce the prevalence of behaviors linked to disease and mortality [29]. People who identify as “fat” or “obese” have long received stigmatizing “fat-shaming” messages from healthcare providers and public health officials. Stigmatization of people with obesity often has the opposite effect and is ethically indivisible given the stress and emotional and dignitary harms it causes [30]. This further emphasizes the importance of developing a nuanced understanding of the potential impact of a food addiction model on stigma, particularly given that it could have divergent effects on externalized stigma towards others and internalized stigma towards the self.

A recent systematic review of nine empirical studies examining the link between food addiction and weight stigma concluded that limited evidence suggests that food addiction explanations may reduce self-blame and stigma from others, but these benefits may be offset by the adverse impact on self-efficacy and eating behaviors [31]. However, the authors also noted that the existing empirical research is sparse and inconsistent. To our knowledge, no scoping reviews have been conducted to examine the ethical and health policy implications of food addiction, or the implications on stigma more broadly beyond weight stigma. The purpose of this scoping review was to highlight what has been published in the food addiction literature and to address these current gaps to help inform current perceptions by identifying the potential ethical, stigma, and health policy implications of food addiction.

## 2. Materials and Methods

This review was conducted as per the Arksey and O’Malley methodological framework for scoping reviews [32]. As part of the development of our research questions and approach, a stakeholder summit was organized in collaboration with the Canadian Obesity Network (CON) involving a broad and diverse group of 18 stakeholders including local knowledge users (i.e., people with experience with obesity-related treatment and food addiction), representatives from national obesity organizations and patient advocacy groups, primary care physicians, and interprofessional research leaders nationally and internationally in food addiction and/or obesity management. Data from a pre-summit online questionnaire exploring perceptions related to the ethical and policy issues of food addiction in obesity informed summit discussions aimed at identifying key themes related to food addiction. The pre-summit online questionnaire was derived from previous studies on food addiction in obesity [33,34] and included open-ended questions regarding the potential implications of food addiction on stigma, ethics, and health policy. The following five stages were completed as part of the scoping review methodology.

*Stage 1: Identifying the Research Questions*

Based on the analysis of themes resulting from the stakeholder summit, this scoping review was conducted to address the following question: What are the potential ethical, stigma and policy implications of food addiction in obesity?

Inclusion/exclusion criteria

We included peer- and non-peer reviewed articles, editorials, commentaries, letters, and replies to authors that met the following criteria:

1. Were published in English;
2. Were human studies;
3. Established a link between obesity or binge eating and addiction or substance use;
4. Presented a conceptual or mechanistic model of food addiction (i.e., had to include terms such as “reward,” “reward pathway,” “compulsion,” “substance use,” or “pleasurable” somewhere in the article); and
5. Presented the issue of food addiction using a key concept (rather than quoting a prevalence).

We excluded book chapters, dissertations, and policy documents. Papers were also excluded if they only focused on obesity and did not relate to food addiction.

*Stage 2: Identifying Relevant Studies*

We searched for relevant articles published up until January 26, 2017, for each question using the following databases: Medline and PsycINFO from the OvidSP platform and CINAHL from the EBSCO platform. Each of the OvidSP databases was searched individually. The searches were conducted with assistance from a research librarian from an academic health sciences center. The searches were run with the limits of human studies and English language studies only. When appropriate, controlled vocabulary terms and/or text words were used in the subject component blocks. For example, obesity/bariatric terms included one block, food addiction terms included a second block, and ethics/stigma/policy terms included a third block. Lastly, we reviewed key special issues on food addiction in prominent eating disorder and obesity periodicals to identify additional articles that may have met our inclusion criteria and were missed by our initial search. A total of 410 articles were identified from these search strategies.

*Stage 3: Selecting Studies*

**Figure 1:**
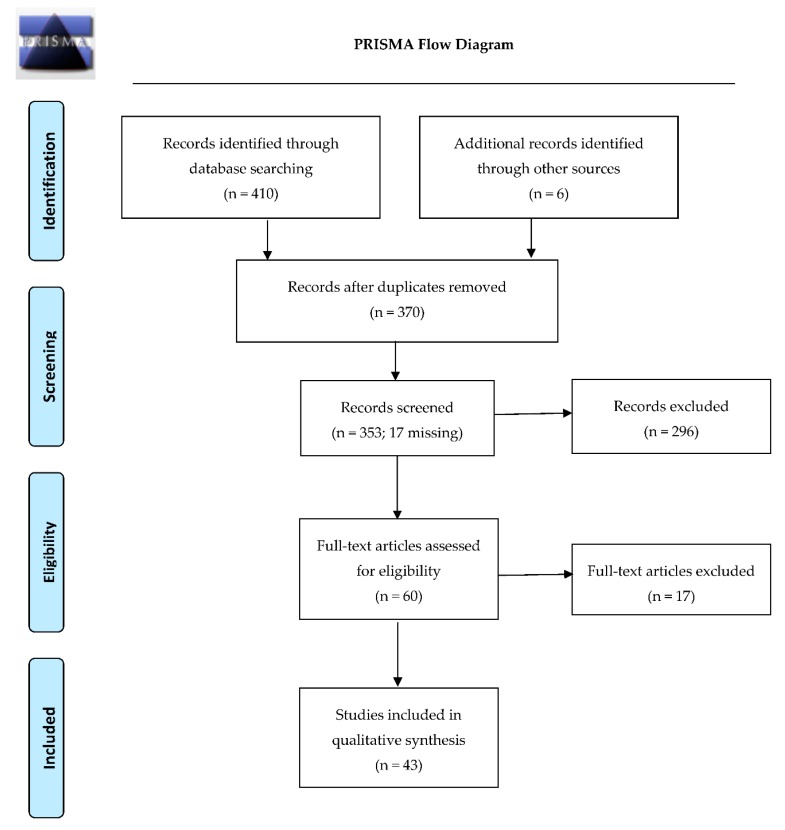
PRISMA flow diagram depicting the flow of information through the different phases of this scoping review. From: [35].

All references resulting from the literature search were reviewed and 46 duplicates were removed. Preliminary screening was conducted by titles and abstracts by two independent reviewers. Seventeen references were missing and were excluded from screening. Screened abstracts were separated into three categories: articles to include, unsure articles, and non-relevant articles to exclude. A secondary screening was then conducted by two additional independent reviewers on the articles that were deemed unsure from the first round of screening. This screening procedure resulted in 296 articles being excluded. Full text articles for the remaining 60 abstracts were then retrieved for data collection (see Figure 1 for PRISMA diagram).

*Stage 4: Charting the Data*

Data were extracted from all included full-text articles by one reviewer into a spreadsheet that included authors, year of publication, and article type and relevant excerpts, themes, and quotes. Each domain was then separated into individual spreadsheets with the relevant articles and their respective identifiers and variables for coding and analysis.

*Stage 5: Collating, Summarizing, and Reporting the Results*

Three independent reviewers completed this step. Each of the three main domains of ethics, stigma, and policy had two reviewers who independently reviewed the full text articles and coded the data. A thematic analysis approach was used to critically analyze the articles and generate open codes. All reviewers then came together to discuss, compare, and organize the relevant excerpts, themes, and quotes that were extracted for each of the three main domains. These iterative discussions allowed for a higher level of thematic analysis, generating broad categories and higher-level themes. Reviewers met nine times at 1- to 2-month intervals for a total of 18 h to review codes and to generate domains summarized below.

## 3. Results

Each of the three domains of ethics, stigma, and policy implications related to food addiction are described below. All articles included from the search strategy for each of the three domains are listed in Table S1. Of the 43 studies included in the analysis, 10 were reviews, 25 were original research articles, 5 were commentaries, 2 were opinions, and 1 was a brief. Table 1 provides a summary of themes and subthemes with sample quotes extracted from articles.

### 3.1. The Intersection of Ethics and Food Addiction

Our search yielded 20 articles related to ethics and food addiction. We identified individual responsibility as the primary theme of this section, with the following sub-themes: (i) personal control, will power, and choice; and (ii) blame and weight bias. 

#### 3.1.1. Personal Control, Will Power, and Choice

We found a tension in the literature between the traditional framings of addiction that emphasized personal control over food consumption, with medicalized, neuroscientific accounts of food addiction that emphasized reduced personal control. For example, Appelhans, Whited, Schneider, and Pagoto suggested, “[d]ietary lapses or failures should be conceptualized as the result of brain systems interacting with a toxic food environment, and not as a reflection of poor personal choices or lack of willpower” [45] (p. 1135). Some scholars argue that framing food addiction within a disease model may reduce individual blame [46] as the impairment in the person’s autonomy and capacity for control reflects dysfunction in the brain’s reward circuitry [36,44,47]. However, some scholars are skeptical that a food addiction label or a disease model will reduce responsibility or obviate perceptions that obesity is self-inflicted [5,48]. Others worry about the potential negative influence a brain disease model of food addiction might have on people with obesity. For example, Lee and colleagues suggest that a (brain) disease model could potentially justify coercive practices on a population that is thought to have reduced autonomy and limited control over their behavior [33].

A study by Lee and colleagues found that public support for a food addiction model of obesity was associated with the view that the individual had to overcome their condition through willpower and personal choice [34]. Other studies have reported that the public considers food addiction more of a personal choice than an addiction to other substances such as alcohol [41]. Green suggests that the free autonomous behavior to consume highly palatable foods could be deterred potentially by the implementation of sin taxes (or excise taxes) on said foods [49], but others, such as Franck, Grandi, and Eisenberg, question the evidence to support this recommendation [50]. Scholars have argued that the focus on individual-level factors, such as free will and personal choice, have failed to address the public health impact of high levels of highly palatable food consumption [51,52]. Some authors have argued that this narrow focus on individual-level factors “lets the food industry off the hook” for its contributions [53] (p. 2).

#### 3.1.2. Blame and Weight Bias

Given the emphasis on personal responsibility in the literature, perspectives on who or what is to blame for food addiction are also far from stable. The literature reflects tensions between attributing blame for food addiction to the individual, the environment (including government and industry), or both, but seldom the alleged addictive properties of the food itself [5,41]. For instance, Thibodeau, Perko, and Flusberg found that participants who agreed with narratives that blamed the individual for their obesity (i.e., due to an addiction, a problem of individual behavior) were likely to support interventions that were penalizing, whereas support for narratives that blamed the environment were likely to support policy interventions that sought to protect people with obesity [37]. These tensions exist in a Western culture that values and legitimizes the over-consumption of foods and other material goods [51,54].

Weight bias is an ethically important concept in terms of its relevance to respect for persons, discrimination, and social justice, among others [38]. For example, entrenched harmful stereotypes that describe people with obesity as lazy and lacking in willpower underpins the moral view that they make poor choices with respect to food consumption and their health [38]. “Fat” people are considered “addict[ed] to inappropriate pleasures,” which serves to further stigmatize and blame “fat” people [55] (p. 17).

### 3.2. Potential Implications of Food Addiction on Stigma

Our search yielded 19 articles related to stigma and food addiction. Identified themes included the following: (i) the impact on self-stigma and stigma from others, (ii) the differential impact of substance use disorder versus behavioral addiction on stigma, and (iii) the additive stigma of addiction plus obesity and/or eating disorder. As noted below, there was extensive overlap between several of the stigma and ethics themes.

#### 3.2.1. The Impact on Self-Stigma and Stigma from Others

Related to the concepts of personal control, willpower, and choice, as well as blame and weight bias described above, several authors discussed potential implications of food addiction on self-stigma (internalized stigma) and stigma from others (externalized stigma). Similar to people who use substances, people with obesity receive messages from individuals and society that their obesity is a character flaw [56]. Empirical research using hypothetical case vignettes has found some evidence to suggest that a food addiction model may have a beneficial impact on stigma towards others who are overweight. Latner, Puhl, Murakami, and O’Brien randomly assigned participants to one of four conditions in which they read about either an addiction or non-addiction explanatory model of obesity, and subsequently read a vignette about a woman that was either obese or of normal weight [46]. Regardless of the participant’s weight status, a food addiction explanatory model of obesity was found to result in less stigma, less blame, and less perceived psychopathology attributed to the woman in the vignette [46]. Moreover, a food addiction model resulted in less blame towards individuals with obesity in general, leading the authors to conclude that this explanatory model of obesity could have a beneficial impact on the pervasive prejudice against individuals with obesity.

Lee et al. conducted a large survey regarding public views of food addiction [34]. They found substantial support for the concept of food addiction, particularly among individuals with obesity [34]. They were less likely to agree that obesity is caused by overeating and they reported greater support for external causes of obesity. The authors speculated that a diagnosis of food addiction may reduce some of the guilt and self-blame associated with obesity. However, a correlational study by Burmeister, Hinman, Koball, Hoffman, and Carels found that weight-loss-seeking participants with a greater severity of food addiction, as measured by the YFAS, reported greater anti-fat attitudes (e.g., dislike of other people with excess weight), internalized weight bias (e.g., internalization of anti-fat attitudes), and body shame [39].

#### 3.2.2. Differential Impact of Substance Use Disorder Versus Behavioral Addiction on Stigma

Despite evidence of shared neurobiological processes in substance use disorders and behavioral addictions, a false dichotomy permeates much of the discourse whereby substance use disorders are perceived as a neurobiological disease and behavioral addictions are perceived as a mental/psychological condition. Whether food addiction is considered a substance use disorder versus behavioral addiction may impact factors such as personal responsibility, autonomy, and blame attribution, which in turn have implications for internalized and externalized stigma. Stigma may decrease if food addiction is considered a substance use disorder because it provides an explanatory model for obesity “without invoking character flaws, such as lack of willpower” [45] (p. 1133), and shifts responsibility from the individual to the food substance and food industry. For example, Appelhans et al. state: “by emphasizing genetically-influenced neurobiological processes that confer vulnerability to overeating in a toxic food environment, the model enables dietetics practitioners to more effectively address obesity without promoting stigma” [45] (p. 1133). However, Rasmussen points out that the “neurochemical concept of obesity, as with drug addiction and eating disorders, can coexist with attribution of personal responsibility for the condition, and thus with blaming the impaired, addicted consumer rather than the supplier” (p. 217), and goes on to say that “it cannot be ruled out that attribution of addiction (as a chronic brain disease) will hurt more than it helps” [5] (p. 223).

Whereas opinions were mixed regarding whether considering food addiction as a substance use disorder would reduce internalized and externalized stigma, it was generally believed that considering food addiction as a behavioral addiction would either have little effect on stigma or actually increase stigma because it is perceived as a choice that is under personal control. DePierre, Puhl, and Luedicke conducted an empirical study to examine public perceptions of food addiction compared to nicotine and alcohol addiction. Food addiction was considered to be a disease to a greater extent than smoking and to be the product of personal choice to a greater extent than alcohol addiction, leading the authors to conclude that food addiction may be perceived as a behavioral addiction that is vulnerable to stigmatization rather than a substance addiction [41].

#### 3.2.3. Additive Stigma of Addiction Plus Obesity/Eating Disorder

We found a tension in the literature regarding whether food addiction would reduce the stigma directed towards individuals with obesity or create an additional stigmatized identity. Several authors pointed out that substance-related disorders, such as nicotine, alcohol, and cocaine use disorders, are stigmatized to an even greater extent than obesity, a finding that may be due to the fact that everyone must eat food for survival, whereas a person must choose to seek out and use psychoactive substances of abuse [40,41,45]. It has also been suggested that food may be a less stigmatizing substance of abuse because it has less impact on others compared to nicotine (e.g., second-hand smoke) and alcohol (e.g., driving under the influence, impulsive behaviors) [40,41].

Several authors raised concerns that a diagnosis of food addiction could result in a double or additive stigma because the types of stigma associated with obesity and addiction differ [5,40,42,57]. For example, applying the types of stigma described by Goffman in this context, obesity could be considered a visible “abomination of the body,” whereas addiction could be considered a “blemish of character” [40,58]. Foddy states: “I have said nothing about the myriad social dimensions in which we stigmatize and disadvantage addicts [sic] differently from the obese. It is hard to say exactly which group has the worse lot in this sense, since the stigma and discrimination takes different forms in each case” [36] (p. 87).

Rather than food addiction reducing stigma by providing an explanatory model for compulsive/binge eating and obesity that reduces blame and personal responsibility, several authors noted that food addiction may actually amplify the harms of stigma because obesity, eating disorders, and addictions are all stigmatized conditions, and neurobiological explanations do not necessarily modify attributions of personal responsibility. Allen et al. raised concerns about the “dietary obese being stigmatized as addicts [sic]” (p. 134) [42]. Rasmussen noted that “the stigmatizing attribution of addiction to people already stigmatized as obese may amplify the social harms that they suffer” (p. 217), “raising the risk that their combination might prove reinforcing” [5] (p. 223).

A few experimental studies have been conducted to examine the impact of food addiction on externalized stigma. Bannon, Hunter-Reel, Wilson, and Karlin randomly assigned participants to one of six conditions in which they read a vignette about a woman with obesity [59]. The independent variables were her binge eating status (present vs. absent) and the cause of her obesity (biological addiction vs. psychological vs. ambiguous). Unfortunately, the manipulation regarding the cause of her obesity was not successful, which prevented interpretation of the specific impact of food addiction on externalized stigma. However, the presence of binge eating increased externalized stigma such that participants rated individuals with obesity as less attractive and more blameworthy for their weight, and they desired greater social distance from them [59]. DePierre et al. directly examined the additive effect of food addiction on externalized stigma [40]. They found that labelling an individual as an “obese food addict” was more stigmatizing than either label on its own (“food addict” or “obese”) and concluded that food addiction may increase the externalized stigma associated with obesity, but be less susceptible to stigma than other forms of addiction [40].

### 3.3. Potential Policy Implications of Food Addiction

Our search yielded 31 articles related to policy themes for addictive foods that were extrapolated to the construct of food addiction. Policy themes related to addictive foods were generally derived from comparisons to the tobacco industry. Articles identified specific policy interventions from “Big Tobacco” that could be applied to food addiction included the following: (i) reducing access to addictive foods, (ii) food taxation, and (iii) limiting advertising for addictive foods.

#### 3.3.1. Comparisons to “Big Tobacco”

Many articles made parallels between the policy issues related to the food industry and the tobacco industry. The role of food taxation, reducing access to potentially addictive foods, litigation against the food industry, and limiting food advertising in addressing food addiction and obesity arise from this comparison to “Big Tobacco” and highlight a prominent theme amongst most articles focusing on policy issues of food addiction [38,52,53]. The association between the food industry and tobacco industry is contingent on a clear link between certain foods and their addictive potential. Authors argued that if certain foods are conceptualized as “a potentially disease-causing agent such as tobacco and alcohol then policy changes to encourage the intake of healthy food and decrease the intake of unhealthy foods are in order” [60] (p. 762). Furthermore, Schulte and colleagues argue that if the concept of food addiction is embraced by the public as it was for nicotine addiction, the shift in public perception of the substance (in this case, addictive foods) could generate enough support for change in public policies for obesity [61]. This argument has been supported by an online survey of 999 individuals from the U.S., including 52% of individuals who were overweight or obese, which showed that believing food is addictive increased support for obesity-related policies [62]. 

#### 3.3.2. Reducing Access to Addictive Foods Through Regulation

In comparison to policies in the tobacco industry, the overall evidence for the effect of government regulations on limiting access to highly pleasurable food is still emerging and requires further exploration [63]. Current papers identified the role of government regulations on the food industry to support legislation, litigation, and regulation efforts to influence access to healthy as opposed to high fat or sugar foods [60,64]. Reports suggest that if food is deemed addictive, then governments need to establish clear policies to limit, restrict, or even ban addictive foods [52], such as limiting the number and location of fast food restaurants and soft drink vending machines in an area.

The support for policies focused on addictive food restriction for children and youth as opposed to adults. For example, in a survey of youth who were unable to lose weight using an online open-access website for obesity, participants felt that restriction of junk food and fast food outlets would be beneficial [36]. Additional articles supported early intervention efforts through increased regulation of pleasurable foods in children and youth settings such as schools, implementation of programs promoting healthy eating and food choices, and engagement of pediatricians and family physicians [65,66]. In contrast, a recent qualitative study with 23 individuals with obesity showed that individuals were skeptical about the effectiveness of restrictions on highly pleasurable foods, although they supported addictive food restrictions for children [43]. Nonetheless, reviews generally supported external control of addictive foods based on examples where cigarette smokers perceived external control as being influential or effective compared to individual motivating factors (e.g., willpower, personal choice) [67,68].

Despite this support for policy interventions focused on increased food regulation, concerns were expressed in articles around the lack of clarity around “addictive,” or “good” and “bad,” ingredients and foods [69]. The lack of clear evidence about foods categorized as “addictive” and the unique challenges with food being a necessary part of living (in contrast to alcohol) have complicated abstinence arguments and approaches thus far. Nonetheless, authors have used the comparison to the tobacco industry for informing food addiction policy interventions focused on restriction [56]. Empirical evidence has demonstrated that narratives where obesity was considered an addiction or a disorder led to increased public support for a protective approach to policy interventions for obesity; however, implications for obesity treatment are not fully understood [37].

Studies reiterated the importance of obesity experts being more explicit about food engineering and the need for greater public awareness of this issue beyond the traditional “obesogenic environment” discourses [44]. A greater focus on increasing public awareness about food engineering could lead to greater support for policy interventions focused on regulation and addictive food restriction. One article noted that a potential consequence of identifying and regulating specific “addictive” foods could be litigation and banning of the food industry for purposefully engineering foods to be more addictive [52].

#### 3.3.3. Food Taxation

Themes related to food taxation also emerged predominantly from comparisons between the food and tobacco industries, as highlighted above [42,44]. The rationale stems from evidence for the taxation of tobacco products, which has estimated that a 10% increase in soft drink prices could result in approximately an 8–10% reduction in soft drink consumption by individuals [70,71]. Moreover, several countries, including France and Hungary, have implemented tax levies on high caloric density foods [41]. These national policy initiatives will be beneficial in evaluating the effects of these interventions longitudinally on mitigating food addiction.

Despite this early evidence, several factors complicate food taxation as a policy intervention to reduce access to caloric dense foods. In contrast to tobacco products, caloric dense foods are already cheaper than healthier food options [42]. Therefore, food taxation efforts would affect children and the poor disproportionately given the current low cost of caloric dense foods, although food taxes may potentially be used to offset costs for healthier foods in this population [42]. In addition, it is unclear what food ingredients are highly addictive and as a result, conclusive recommendations on specific ingredients warranting taxation remains a challenge [53].

#### 3.3.4. Limiting Advertising for Addictive Foods

Studies have also articulated concerns about the early introduction of food advertising to young children and adolescents. Gearhardt and colleagues identified the need to limit food advertising to children to limit the potential long-term effects, such as food addiction [38]. Restriction of food marketing to youth has involved both government policies but also voluntary pledges by the food industry [38]. These initiatives have attempted to correct the default food industry messaging that has been inoculating children and youth for decades [71]. As a result, authors argue that voluntary self-regulatory efforts by the food industry are likely to be insufficient, and government protective policies are needed to counteract these trends in early childhood food marketing strategies by the food industry [38].

#### 3.3.5. Limited Information on Food Addiction in Obesity Guidelines

Several papers focused on the limited discussion of food addiction within key obesity related guidelines and resources such as the World Health Organization (WHO) and Centre for Disease Control (CDC) [42]. Despite increasing public dialogue on food addiction, obesity guidelines have not included information on the controversies related to food addiction. This is complicated by the inability to differentiate specific addictive food ingredients and limited evidence for food addiction related interventions [72]. As a result, papers highlighted the need for an improved mechanistic understanding and the development and testing of new treatments for managing addictive foods.

Furthermore, there have been questions regarding the evidence for policy interventions for preventing and managing addictive foods in patients with obesity. Gostin and colleagues believe that the “lack of science” argument for food addiction policy interventions is a faulty argument given that no single intervention resulted in a substantial reduction in nicotine use and there is a need for a multi-level intervention approach [73]. Thus, further research is needed to clarify the construct of food addiction, risk factors and effectiveness of policy interventions to facilitate integration within obesity-related guidelines and resources.

## 4. Discussion

The current scoping review examined the potential ethical, stigma, and health policy implications of food addiction, identified the major themes in the literature, and provided insights into key challenges in these areas.

### 4.1. Potential Ethical Implications of Food Addiction

While various disease models of alcohol use disorders have circulated within the popular discourse since the early 19th century [74,75], more recently scholars have adopted the language of neuroscience to characterize addiction more generally as a chronic, relapsing brain disease [76,77]. We found that discussions in the literature lend themselves to moral debates over personal control, individual responsibility, and blame for the consequences of food consumption. Indeed, these moral framings are common in the obesity discourse [44,78]. However, the neurobiological theory of food addiction may place a disproportionate burden on the individual to treat their food addiction, which may provide less incentive for governments to scale up public health approaches, such as holding the food industry accountable for their food engineering practices as well as the social conditions that harm people who are defined as food addicted.

The focus of individual blame in the literature may be reflective of a culture of healthism, defined as the individual desire for health and well-being achieved primarily through lifestyle modification [79]. The moral assumptions underlying healthism is that individuals are responsible for making good choices as opposed to bad choices about their health. Our results suggest that healthism describes good choices as health-promoting and reflects self-control over eating while bad choices lead to food addiction or obesity. For instance, some dietary counselling recommendations include empowering the patient to take responsibility for their behaviors in the context of what they consider to be a toxic food environment [45]. Our results suggest that some authors were concerned about a culture that emphasizes healthism and individualism when the social conditions make the ability to make healthy eating choices near impossible [51]. Future research can build on the burgeoning literature in this area and provide deeper insights into the moral attitudes about food addiction held by diverse publics. It will also be important for future research to explore if the empirical findings related to moral concepts align with the concerns raised in the conceptual philosophical and bioethics literatures.

### 4.2. Potential Stigma Implications of Food Addiction

Our review also examined the complex relationship between food addiction and stigma. Stigma is a multidimensional construct including many related concepts (e.g., labels, stereotypes, prejudice, discrimination), sources (e.g., self-stigma, public stigma, provider-based stigma [e.g., from healthcare providers], institutional stigma), characteristics (e.g., stigma of the physical body, stigma of character), and dimensions (e.g., social distance, perceptions of dangerousness) that can complicate analyses [80]. The limited empirical research conducted to date, much of which has experimentally manipulated the information provided in case vignettes, has generated mixed results regarding the impact of a food addiction model on externalized stigma. Whereas a food addiction explanation for obesity was found to reduce externalized stigma and blame towards a target in one study [45], another study found that labeling an individual as an “obese food addict” created an additive stigma that exceeded the stigma associated with either label on its own [40]. This finding is consistent with a recent qualitative study of individuals with obesity, which noted that some individuals who personally identified with the concept of food addiction felt reluctant to be described as an “addict” because they believed the label would increase self-stigma and stigma from others [43]. Interestingly, the one study identified through our scoping review that examined the association between food addiction and externalized stigma in a clinical sample found that patients with greater severity of food addiction reported greater anti-fat attitudes towards other individuals with excess weight [39]. The impact of food addiction on externalized stigma has been examined in student samples, community samples, and a clinical sample, and additional research is warranted to examine the impact of a food addiction model on externalized stigma among healthcare professionals.

In contrast to the mixed findings regarding the impact of a food addiction model on externalized stigma, the association between food addiction and internalized stigma has been fairly consistent. The aforementioned study conducted in a clinical sample found that patients with greater severity of food addiction reported greater internalized weight bias and body shame, as well as lower eating self-efficacy [39]. This finding is consistent with a recent study comparing three groups that varied with respect to food addiction (i.e., non-food-addicted, self-perceived food addicted, and food-addicted according to the YFAS) and found that internalized weight bias increased across the groups despite no differences in body mass index, whereas eating self-efficacy decreased across the groups [81]. Similarly, a recent systematic review examining the association between internalized weight bias and health-related variables reported that the published studies conducted to date have consistently found a positive association between food addiction and internalized weight bias [82]. Moreover, internalized weight bias was associated with a variety of negative mental and physical health outcomes including depression, anxiety, low self-esteem, poor body image, disordered eating, severity of obesity, reduced dietary adherence, and reduced motivation and self-efficacy to engage in health-promoting behaviors. Although much of the research conducted to date has been cross-sectional, which precludes conclusions regarding the direction of causality, if replicated in prospective research, such findings would lend support to concerns voiced by some authors that food addiction could have an adverse impact on internalized stigma and reduce the self-efficacy needed to improve eating behaviors [29]. Although the treatment implications of food addiction were beyond the scope of the current review, this important topic is recently receiving more attention in the literature [83,84] and the findings of the current review suggest that treatment approaches that reduce internalized weight bias and bolster self-efficacy would be advised.

Whether food addiction is best characterized as a behavioral addiction or a substance use disorder has been a topic of debate [26]. The results of the current review suggest that characterizing food addiction as a behavioral addiction likely has an adverse impact on externalized stigma compared to characterizing it as a substance use disorder. Despite evidence that behavioral addictions and substance use disorders have many shared features, including neurobiological mechanisms and genetic contributions [85,86], behavioral addictions are still commonly perceived as being more under personal control. The general public perceives food addiction as being less of a disease and more under personal control than alcoholism [41]. Increasing public awareness regarding the engineering of hyperpalatable foods and the validity of food addiction as a diagnostic construct that shares similarities with other substance use disorders [8] may be helpful as both a public health and stigma reduction intervention.

### 4.3. Potential Policy Implications of Food Addiction

Across reviewed articles, we found that the tobacco industry served as an example for many of the policy recommendations for managing food addiction in obesity. The challenges with these policy interventions were related to the lack of research on addictive food substances, specifically the current inability to classify foods more broadly in their addictive potential. Despite authors drawing parallels to “Big Tobacco,” we identified a dearth of evidence for the effectiveness of food addiction as a driver for policy change in obesity. Therefore, many of the recommendations, such as food taxation, reducing advertising to children, and reducing access to addictive foods (especially in children and youth), were extrapolated from policy interventions observed in the tobacco industry as opposed to being specific to food addiction.

### 4.4. Limitations

Our results are subject to the following limitations associated with scoping review. Unlike a systematic review, we are unable to grade the quality of the studies or make any specific recommendations related to level of evidence. The articles included in this scoping review included commentaries and empirical research (e.g., experimental and correlational studies). Further, although our search used multiple methods to identify potential articles, it is possible that some studies were missed. This scoping review underscored the need for future research on the effectiveness of policy interventions to mitigate the effect of addictive foods on the obesity epidemic. The inclusion of food addiction within obesity guidelines was a notable omission in the literature that was identified in our review. It is possible that the absence of food addiction in obesity guidelines reflects the need for greater research on the role of food addiction on obesity management, including questions about its benefit for healthcare professionals and patients in obesity care.

## 5. Conclusions

This review is the first to explore the ethical, stigma, and policy issues related to food addiction using a scoping review methodology. The gaps in the evidence related to this scoping review underscore the need for additional research and increased clarity regarding the ethical and stigma related impact of a food addiction model. In addition, more rigorous studies are needed to move beyond policy interventions generated predominantly from past experiences in the tobacco industry.

## Figures and Tables

**Table 1:** Scoping review themes, subthemes, and representative quotes.

Themes and Sub-Themes	Authors	Sample Quote
**ETHICS**		
Personal control, will power, and choice	Foddy (2011) [36]	“The disease label implies a reduction in the autonomy of the addicted and obese. If their strong preferences for consuming food or drugs are merely the symptoms of disease, then these preferences should be viewed as irrelevant in assessing whether their behaviour is willful or not” (p. 86).
	Lee et al. (2014) [33]	“Equating obesity with food addiction could even justify the use of coercive treatments if obese individuals are seen to suffer from a form of addiction over which they have limited control” (pp. 5313–5314).
Blame and weight bias	Thibodeau et al. (2015) [37]	“The narrative of ‘addiction’ attributed relatively more blame to the behavior of an [obese] individual, evoking comparisons to alcoholism or drug abuse” (p. 29).
	Gearhardt et al. (2012) [38]	“[I]n addition to causing individual suffering weight bias creates personal and societal injustices for obese persons” (p. 409).
**STIGMA**		
Impact on self-stigma and stigma from others	Burmeister et al. (2013) [39]	“Food addiction symptoms were related to self-reports of internalized weight bias as well as a form of explicit weight bias in which participants indicated dislike of people who carry excess weight and fears related to becoming or remaining overweight. Given our culture’s traditional views of addiction being a blameworthy illness, it may come as no surprise that obese food ‘addicts’ might tend to internalize these stigmatizing beliefs (p. 108).”
Differential impact of substance use disorder versus behavioral addiction	DePierre et al. (2013) [40]	“From the perspective of attribution theory, applying a food addict label to an obese individual could either ameliorate or exacerbate weight stigma. Attribution theory posits that the more a person is seen as responsible for his or her condition, the more people will blame and react to him or her negatively. Indeed, ascribing the cause of obesity to behavioral factors within personal control has been demonstrated to increase stigma, whereas people display fewer negative attitudes toward an individual whose overweight is attributed to biogenetic or physiological factors outside of personal control. Food addiction could be perceived as an external explanation for obesity, reducing blame and stigma. Alternatively, a food addict label could instead act as a behavioral causal attribution, leading obesity to be perceived simply as a result of overeating, potentially increasing weight bias (p. 11).”
	DePierre et al. (2014) [41]	“Food addiction was perceived as a problem of the mind more than either smoking or alcoholism, and received high endorsement as a behaviour resulting from personal unhappiness, indicating that food addiction is viewed as a mental or behavioural problem rather than a physical addiction. Such a perception may detract from beliefs that certain foods can be addictive, with food addiction instead being seen as rooted in an individual’s psychological make-up. Additionally, given that mental illnesses elicit more stigma and blame than physical ailments, it is possible that food addiction may increase bias towards overweight/obese individuals with this disorder (p. 5).”
Additive stigma of addiction plus obesity and/or eating disorder	DePierre et al. (2013) [40]	“In the context of attribution theory, the food addict label may have increased blame toward obese individuals by attributing weight to eating behavior, where food addiction may be interpreted as a euphemism for overeating. Or perhaps, in line with Goffman’s (1963) framework, categorizing obesity as the result of an addiction added to this ‘abomination of the body’ the stigma of a ‘blemish of character’. Perceiving obesity as the result of a personal failing such as addiction may extend the domains in which it is stigmatized from the immediate social interaction to perceptions of competency for more solitary tasks (or, obesity may extend the otherwise concealable stigma of food addiction to social interactions), potentially explaining why an obese food addict was more negatively perceived than a food addict alone (p. 18).”
**POLICY**		
Comparison to “Big Tobacco”	Allen et al. (2012) [42]	“It is also vital to consider the numerous elements unique to the obesity epidemic that may require novel policy strategies. Most evident is the fact that food is essential for survival, whereas tobacco use is viewed as a dispensable, or even recreational, activity.”
Reducing access to addictive foods through regulation	Cullen et al. (2017) [43]	“[Restrictions] would probably help but if the person really wants a Coke every day, they would probably find the means to get it.”
Food taxation	Pretlow RA. 2011 [44]	“Taxation of sugar-sweetened beverages, and possibly junk food and fast food, and restriction of such outlets to children, would seem warranted and even embraced by some children.”
Limiting advertising for addictive foods	Gearhardt et al. (2012) [38]	“[The food industry] has pledged to remove its unhealthy products from schools and to market fewer unhealthy foods to children, in addition to a variety of other promises. Whether the public and elected officials find the industry trustworthy will determine in part how aggressive government will be in regulating industry practices.”
Limited information on food addiction in obesity guidelines	Allen et al. (2012) [42]	“In these guidelines, nothing is said about the potential that an unhealthy diet may not be easily changed due to addictive aspects of hyperphagia. Furthermore, while they do recommend using interventions like taxation on high-energy food, they do not discuss more strict strategies like limiting the access of children to such food.”

## Supplementary Materials

The following are available online at https://www.mdpi.com/2072-6643/11/4/710/s1, Table S1: Complete List of Articles from Search Strategy.
