# Supplementary material for: Ethical, Stigma, and Policy Implications of Food Addiction: A Scoping Review
Source: Nutrients. 2019 Mar 27;11(4):710. doi: 10.3390/nu11040710 (PMC6521112; doi:10.3390/nu11040710)
Supplement: Supplementary file 1 [file nutrients-11-00710-s001.pdf]

Table S1. Complete List of Articles from Search Strategy.

Articles from search strategy included in Ethics section (in alphabetical order):

1. Appelhans, B.M.; Whited, M.C.; Schneider, K.L.; Pagoto, S.L. Time to abandon the notion of personal choice in dietary counseling for obesity? *J Am Diet Assoc* **2011**, *111*, 1130-1136.
2. Blundell, J.E.; Finlayson, G. Food addiction not helpful: the hedonic component - implicit wanting - is important. *Addiction* **2011**, *106*, 1216-1218.
3. Brownell, K.D.; Schwartz, M.B.; Puhl, R.M.; Henderson, K.E.; Harris, J.L. The need for bold action to prevent adolescent obesity. *J Adolesc Health* **2009**, *45*, S8-S17.
4. DePierre, J.A.; Puhl, R.; Luedicke, J. Public perceptions of food addiction: A comparison with alcohol and tobacco. *J Subst Use* **2014**, *19*, 1-6.
5. Foddy, B. Addicted to Food, Hungry for Drugs. *Neuroethics* **2011**, *4*, 79-89.
6. Franck, C.; Grandi, S.M.; Eisenberg, M.J. Taxing Junk Food to Counter Obesity. *Am J Public Health* **2013**, *103*, 1949-1953.
7. Gearhardt, A.N.; Bragg, M.A.; Pearl, R.L.; Schvey, N.A.; Roberto, C.A.; Brownell, K.D. Obesity and public policy. *Annu Rev Clin Psychol* **2012**, *8*, 405-430.
8. Green, R. The Ethics of Sin Taxes. *Public Health Nurs* **2011**, *28*, 68–77.
9. Hebebrand, J. Obesity prevention: Moving beyond the food addiction debate. *J Neuroendocrinol* **2015**, *27*, 737-738.
10. Ho, A.L.; Sussman, E.S.; Pendharkar, A.V.; Azagury, D.E.; Bohon, C.; Halpern, C.H. Deep brain stimulation for obesity: rationale and approach to trial design. *Neurosurg focus* **2015**, *38*, E8.
11. Latner, J.D.; Puhl, R.M.; Murakami, J.M.; O'Brien, K.S. Food addiction as a causal model of obesity. Effects on stigma, blame, and perceived psychopathology. *Appetite* **2014**, *77*, 77-82.
12. Lee, N. M., Lucke, J., Hall, W. D., Meurk, C., Boyle, F. M., & Carter, A. Public Views on Food Addiction and Obesity: Implications for Policy and Treatment. *PLoS One* **2013**, *8*, e74836.
13. Lee, N.M.; Hall, W.D.; Lucke, J.; Forlini, C.; Carter, A. Food Addiction and Its Impact on Weight-Based Stigma and the Treatment of Obese Individuals in the U.S. and Australia. *Nutrients* **2014**, *6*, 5312-5326.
14. Mackenzie, R. Don't Let Them Eat Cake! A View From Across the Pond. The *Am J Bioeth* **2010**, *10*, 16-18.
15. Ortiz, S.E.; Zimmerman, F.J., Gilliam, F.D., Jr. Weighing in: the taste-engineering frame in obesity expert discourse. *Am J Public Health* **2015**, *105*, 554-559.
16. Pomeranz, J.L.; Teret, S.P.; Sugarman, S.D.; Rutkow, L.; Brownell, K.D. Innovative Legal Approaches to Address Obesity. *Milbank Q* **2009**, *87*, 185–213.
17. Pretlow, R.A. Addiction to highly pleasurable food as a cause of the childhood obesity epidemic: a qualitative Internet study. *Eat Disord* **2011**, *19*, 295-307.
18. Rasmussen, N. Stigma and the addiction paradigm for obesity: Lessons from 1950s America. *Addiction* **2014**, *110*, 217-225.
19. Thibodeau, P.H.; Perko, V.L.; Flusberg, S.J. The relationship between narrative classification of obesity and support for public policy interventions. *Soc Sci Med* **2015**, *141*, 27-35.

Articles from search strategy included in Stigma section (in alphabetical order):

1. Allen, P.J.; Batra, P.; Geiger, B.M.; Wommack, T.; Gilhooly, C.; Pothos, E.N. Rationale and consequences of reclassifying obesity as an addictive disorder: neurobiology, food environment and social policy perspectives. *Physiol Behav* **2012**, *107*, 126-137.
2. Appelhans, B.M.; Whited, M.C.; Schneider, K.L.; Pagoto, S.L. Time to abandon the notion of personal choice in dietary counseling for obesity? *J Am Diet Assoc* **2011**, *111*, 1130-1136.
3. Bannon, K.L.; Hunter-Reel, D.; Wilson, G.T.; Karlin, R.A. The effects of casual beliefs and binge eating on the stigmatization of obesity. *Int J Eat Disord* **2009**, *42*, 118-124.
4. Burmeister, J.M.; Hinman, N.; Koball, A.; Hoffmann, D.A.; Carels, R.A. Food addiction in adults seeking weight loss treatment. Implications for psychosocial health and weight loss. *Appetite* **2013**, *60*, 103-110.
5. Criscitelli, K.; Avena, N.M. The neurobiological and behavioral overlaps of nicotine and food addiction. *Prev Med*, **2016**, *92*, 82-89.
6. DePierre, J.A.; Puhl, R.; Luedicke, J. A new stigmatized identity? Comparisons of a 'food addict' label with other stigmatized health conditions. *Basic Appl Soc Psych* **2013**, *35*, 10-21.
7. DePierre, J.A.;Puhl, R.; Luedicke, J. Public perceptions of food addiction: A comparison with alcohol and tobacco. *J Subst Use* **2014**, *19*, 1-6.
8. deShazo, R.D.; Hall, J.E.; Skipworth, L.B. Obesity bias, medical technology, and the hormonal hypothesis: should we stop demonizing fat people? *Am J Med* **2015**, *128*, 456-460.
9. Foddy, B. Addicted to Food, Hungry for Drugs. *Neuroethics* **2011**, *4*, 79-89.
10. Gearhardt, A.N.; Corbin, W.R.; Brownell, K.D. Food addiction: an examination of the diagnostic criteria for dependence. *J Addict Med* **2009**, *3*, 1-7.
11. Latner, J.D.; Puhl, R.M.; Murakami, J.M.; O'Brien, K.S. Food addiction as a causal model of obesity. Effects on stigma, blame, and perceived psychopathology. *Appetite* **2014**, *77*, 77-82.
12. Lee, N.M.; Hall, W.D.; Lucke, J.; Forlini, C.; Carter, A. Food Addiction and Its Impact on Weight-Based Stigma and the Treatment of Obese Individuals in the U.S. and Australia. *Nutrients* **2014**, *6*, 5312-5326.
13. Lee, N.M.; Lucke, J.; Hall, W.D.; Meurk, C.; Boyle, F.M.; Carter, A. Public Views on Food Addiction and Obesity: Implications for Policy and Treatment. *PLoS One* **2013**, *8*, e74836.
14. Mackenzie, R. Don't Let Them Eat Cake! A View From Across the Pond. The *Am J Bioeth* **2010**, *10*, 16-18.
15. Rasmussen, N. Weight stigma, addiction, science, and the medication of fatness in mid-twentieth century America. *Sociol Health Illn* **2012**, *34*, 880-895.
16. Rasmussen, N. Stigma and the addiction paradigm for obesity: Lessons from 1950s America. *Addiction* **2014**, *110*, 217-225.
17. Schulte, E.M.; Tuttle, H.M.; Gearhardt, A.N. Belief in Food Addiction and Obesity-Related Policy Support. *PLoS One* **2016**, *11*, e0147557.
18. Thibodeau, P.H.; Perko, V.L.; Flusberg, S.J. The relationship between narrative classification of obesity and support for public policy interventions. *Soc Sci Med* **2015**, *141*, 27-35.
19. Volkow, N.D.; Wise, R.A. How can drug addiction help us understand obesity? *Nat Neurosci* **2005**, *8*, 555-560.

Articles from search strategy included in Policy section (in alphabetical order):

1. Allen, P.J.; Batra, P.; Geiger, B.M.; Wommack, T.; Gilhooly, C.; Pothos, E.N. Rationale and consequences of reclassifying obesity as an addictive disorder: neurobiology, food environment and social policy perspectives. *Physiol Behav* **2012**, *107*, 126-137.
2. Alonso-Alonso, M.; Woods, S.C.; Pelchat, M.; Grigson, P.S.; Stice, E.; Faroogi, S.; Khoo, C.S.; Mattes, R.D.; Beauchamp, G.K. Food reward system: current perspectives and future research needs. *Nutr Rev* **2015**, *73*, 296-307.
3. Battle, E.K.; Brownell, K.D. Confronting a rising tide of eating disorders and obesity: treatment vs. prevention and policy. *Addict Behav* **1996**, *21*, 755-765.
4. Brownell, K.D.; Schwartz, M.B.; Puhl, R.M.; Henderson, K.E.; Harris, J.L. The need for bold action to prevent adolescent obesity. *J Adolesc Health* **2009**, *45*, S8-S17.
5. Cardin, M.; Farley, T.A.; Purcell, A.; Collins, J. Preventing Obesity and Chronic Disease: Education vs. Regulation vs. Litigation. *J Law Med Ethics* **2007**, *35*, 120–128.
6. Cohen, D.; Farley, T.A. Eating as an Automatic Behavior. *Prev Chronic Dis* **2008**, *5*, A23.
7. Creighton, R. Fat Taxes: The newest manifestation of the age-old excise tax. *J Leg Med* **2010**, *31*, 123-36.
8. Foddy, B. Addicted to Food, Hungry for Drugs. *Neuroethics* **2011**, *4*, 79-89.
9. Franck, C.; Grandi, S.M.; Eisenberg, M.J. Taxing Junk Food to Counter Obesity. *Am J Public Health* **2013**, *103*, 1949-1953.
10. Gearhardt, A.N.; Corbin, W.R.; Brownell, K.D. Food addiction: an examination of the diagnostic criteria for dependence. *J Addict Med* **2009**, *3*, 1-7.
11. Gearhardt, A.N.; Bragg, M.A.; Pearl, R.L.; Schvey, N.A.; Roberto, C.A.; Brownell, K.D. Obesity and public policy. *Annu Rev Clin Psychol* **2012**, *8*, 405-430.
12. Gearhardt, A. N., Dileone, R. J., Grilo, C. M., Brownell, K. D., & Potenza, M. N. Important next steps in evaluating food's addictive potential. *Addiction* **2011**, *106*, 1219-1220.
13. Gearhardt, A.N.; Grilo, C.M.; DiLeone, R.J.; Brownell, K.D.; Potenza, M.N. Can food be addictive? Public health and policy implications. *Addiction* **2011**, *106*, 1208-1212.
14. Gostin, L.O. Limiting what we can eat: a bridge too far? *Milbank Q* **2014**, *92*, 173-176.
15. Green, R. The Ethics of Sin Taxes. *Public Health Nurs* **2011**, *28*, 68–77.
16. Hebebrand, J. Obesity prevention: Moving beyond the food addiction debate. *J Neuroendocrinol* **2015**, *27*, 737-738.
17. Ho, A.L.; Sussman, E.S.; Pendharkar, A.V.; Azagury, D.E.; Bohon, C.; Halpern, C.H. Deep brain stimulation for obesity: rationale and approach to trial design. *Neurosurg focus* **2015**, *38*, E8.
18. Lee, N.M.; Lucke, J.; Hall, W.D.; Meurk, C.; Boyle, F.M.; Carter, A. Public Views on Food Addiction and Obesity: Implications for Policy and Treatment. *PLoS One* **2013**, *8*, e74836.
19. Mercer, A. Obesity, battle of the bulge-policy behind change: Whose responsibility is it and who pays? *Health Educ J* **2010**, *69*, 401-408.
20. Moran, A.; Musicus, A.; Soo, J.; Gearhardt, A. N.; Gollust, S. E.; Roberto, C.A. Believing that certain foods are addictive is associated with support for obesity-related public policies. *Prev Med* **2016**, *90*, 39-46.
21. Ortiz, S.E.; Zimmerman, F.J., Gilliam, F.D., Jr. Weighing in: the taste-engineering frame in obesity expert discourse. *Am J Public Health* **2015**, *105*, 554-559.

22. Pomeranz, J.L.; Teret, S.P.; Sugarman, S.D.; Rutkow, L.; Brownell, K.D. Innovative Legal Approaches to Address Obesity. *Milbank Q* **2009**, *87*, 185–213.
23. Pretlow, R.A. Addiction to highly pleasurable food as a cause of the childhood obesity epidemic: a qualitative Internet study. *Eat Disord* **2011**, *19*, 295-307.
24. Puhl, R.; Suh, Y. Stigma and eating and weight disorders. *Curr Psychiatry Rep* **2015**, *17*, 10.
25. Rasmussen, N. Stigma and the addiction paradigm for obesity: Lessons from 1950s America. *Addiction* **2014**, *110*, 217-225.
26. Schulte, E.M.; Tuttle, H.M.; Gearhardt, A.N. Belief in Food Addiction and Obesity-Related Policy Support. *PLoS One* **2016**, *11*, e0147557.
27. Schwartz, M.B.; Brownell, K. D. Actions Necessary to Prevent Childhood Obesity: Creating the Climate for Change. *J Law Med Ethics* **2007**, *35*, 78–89.
28. Smith, T.G. All foods are habit-forming - what I want to know is which will kill me! *Addiction* **2011**, *106*, 1218-1220.
29. Thibodeau, P.H.; Perko, V.L.; Flusberg, S.J. The relationship between narrative classification of obesity and support for public policy interventions. *Soc Sci Med* **2015**, *141*, 27-35.
309. Volkow, N.D.; Wise, R.A. How can drug addiction help us understand obesity? *Nat Neurosci* **2005**, *8*, 555-560.
31. Willette, A.L. Where have all the parents gone? Do efforts to regulate food advertising to curb childhood obesity pass constitutional muster? *J Leg Med* **2007**, *28*, 561-577.
